# Chronic scrotal content pain and viral orchiepididymitis: an overlooked hypothesis linking infection and Wallerian degeneration

**DOI:** 10.1186/s12610-026-00301-9

**Published:** 2026-03-02

**Authors:** Thiago A. Teixeira, Eduardo Zinoni Pato, Maria Christina W. Avellar, Raul Sanchez, Igor V. Coimbra, Joël R. Drevet, Jorge Hallak

**Affiliations:** 1 Androscience, Science and Innovation Center in Andrology, São Paulo, SP Brazil; 2 Division of Urology, Department of Surgery, University Hospital, School of Medicine, Federal University of Amapá, Macapá, Brazil; 3 Division of Urology, Department of Surgery, Hospital das Clinicas - University of São Paulo Medical School, Sao Paulo, Brazil; 4 Department of Pharmacology, Universidade Federal de São Paulo - Escola Paulista de Medicina, São Paulo, Brazil; 5 Center of Excellence in Translational Medicine–Scientific and Technological Bioresource Nucleus (CEMT–BIOREN), Faculty of Medicine, Universidad de La Frontera, Temuco, Chile; 6 Department of Preclinical Sciences, Faculty of Medicine, Universidad de la Frontera, Temuco, Chile; 7 EVALSEM, GReD Institute, Faculty of Medicine, Université Clermont Auvergne, Clermont-Ferrand, France

**Keywords:** Chronic scrotal content pain, Viral orchiepididymitis, Wallerian degeneration, Male reproductive tract, Chronic neurogenic pain, Semen, Testis, Douleur chronique du Contenu scrotal, Orchido-épididymite virale, Dégénérescence wallérienne, Appareil reproducteur masculin, Douleur neurogène chronique, Sperme, Testicule

## Abstract

**Background:**

Chronic scrotal content pain (CSCP) is a challenging clinical condition, often undiagnosed, and marked by persistent or intermittent pain that may be localized or generalized and significantly affect quality of life. Wallerian degeneration, an inflammatory process involving axonal degeneration, may underlie the mechanism. While bacterial infections are recognized contributors to CSCP, the role of viral infections is less understood.

**Methods:**

We systematically searched PubMed, MEDLINE, and Web of Science for studies reporting associations between viral infections and scrotal or testicular pain. Of 142 identified records, 61 met the inclusion criteria and were analyzed qualitatively.

**Results:**

Our review assesses whether testicular or epididymal viral infections might contribute to CSCP. The literature points to a possible link, though definitive histopathological evidence remains scarce. Tissue biopsies may provide greater diagnostic clarity and inform treatment decisions.

**Conclusions:**

Additional research is needed to understand how viral infections affect testicular function, semen quality, and fertility, to improve patient outcomes globally.

## Résumé

**Contexte:**

La douleur chronique du contenu scrotal est une affection clinique difficile, souvent non diagnostiquée, et caractérisée par une douleur persistante ou intermittente, pouvant être localisée ou généralisée, et affectant la qualité de vie de façon significative. La dégénérescence wallérienne, un processus inflammatoire impliquant la dégénérescence axonale, pourrait être à la base de ce mécanisme. Bien que les infections bactériennes soient reconnues comme contributrices à la douleur chronique du contenu scrotal, le rôle des infections virales est moins compris.

**Méthodes:**

Nous avons systématiquement recherché sur PubMed, MEDLINE et Web of Science, des études rapportant des liens entre infections virales et douleurs scrotales ou testiculaires. Sur les 142 dossiers identifiés, 61 répondaient aux critères d’inclusion ; ils ont été analysés qualitativement.Résultats. Notre revue a évalué si des infections virales testiculaires ou épididymaires pourraient contribuer à la douleur chronique du contenu scrotal. La littérature suggère un lien possible, bien que les preuves histopathologiques définitives restent rares. Des biopsies tissulaires pourraient apporter une plus grande clarté diagnostique, et orienter les décisions thérapeutiques.

**Conclusions:**

Des recherches supplémentaires sont nécessaires pour comprendre comment les infections virales affectent la fonction testiculaire, la qualité du sperme et la fertilité, afin d’améliorer les résultats des patients à l’échelle mondiale.

## Introduction

Chronic scrotal content pain (CSCP) presents as persistent or intermittent scrotal discomfort, frequently limiting daily activities [1–3]. It is common among patients seen by andrologists worldwide, with a reported prevalence of 0.4% to 4.75%, equating to over 100,000 cases annually [4, 5]. Notably, up to half of CSCP cases lack an identifiable cause—frustrating both patients and clinicians and resulting in diagnostic delays, inappropriate therapies, and ongoing symptoms after treatment [6].

CSCP arises from many causes. Intrinsic factors include testicular torsion, trauma, varicocele, hydrocele, epididymitis, orchitis, vasectomy, and complications from surgical procedures. Extrinsic factors comprise lumbar or ureteral disorders, urinary tract stones, inguinal hernia and postoperative changes, and prostate conditions [7, 8]. The underlying biological mechanisms remain complex. One leading concept is that chronic inflammation triggers Wallerian degeneration of neuronal axons, leading to persistent neurogenic pain [4, 8, 9]. This occurs distal to injury or inflammatory sites, reflecting shared nerve pathways. While the contribution of bacterial infection is well established, the effect of viral infection is less defined.

Many viruses inhabit the male reproductive tract and can damage tissue, affecting sperm quality and fertility [10–12]. Here, we review evidence linking viral infection to CSCP and summarize male reproductive anatomy and its somatic and autonomic nerve supply. We propose that viral infection of the testis or epididymis may cause CSCP via chronic inflammation and subsequent Wallerian degeneration. Given the existing criteria for other causes of scrotal pain, we seek to broaden consideration to include possible but less recognized viral origins.

## Materials & methods

We conducted a literature search using PubMed, MEDLINE, and Web of Science for studies published until November 2024 that reported viral associations with scrotal or testicular pain. The defined search strategy included relevant keywords and Boolean combinations such as “viral testicular pain,” “viral orchiepididymitis,” and “chronic scrotal pain.” Only studies in English were considered. Included articles described viral causes of acute or chronic scrotal/testicular pain in humans; studies in non-humans, duplicates, and those lacking clinical or mechanistic relevance were excluded.

**Fig. 1:**
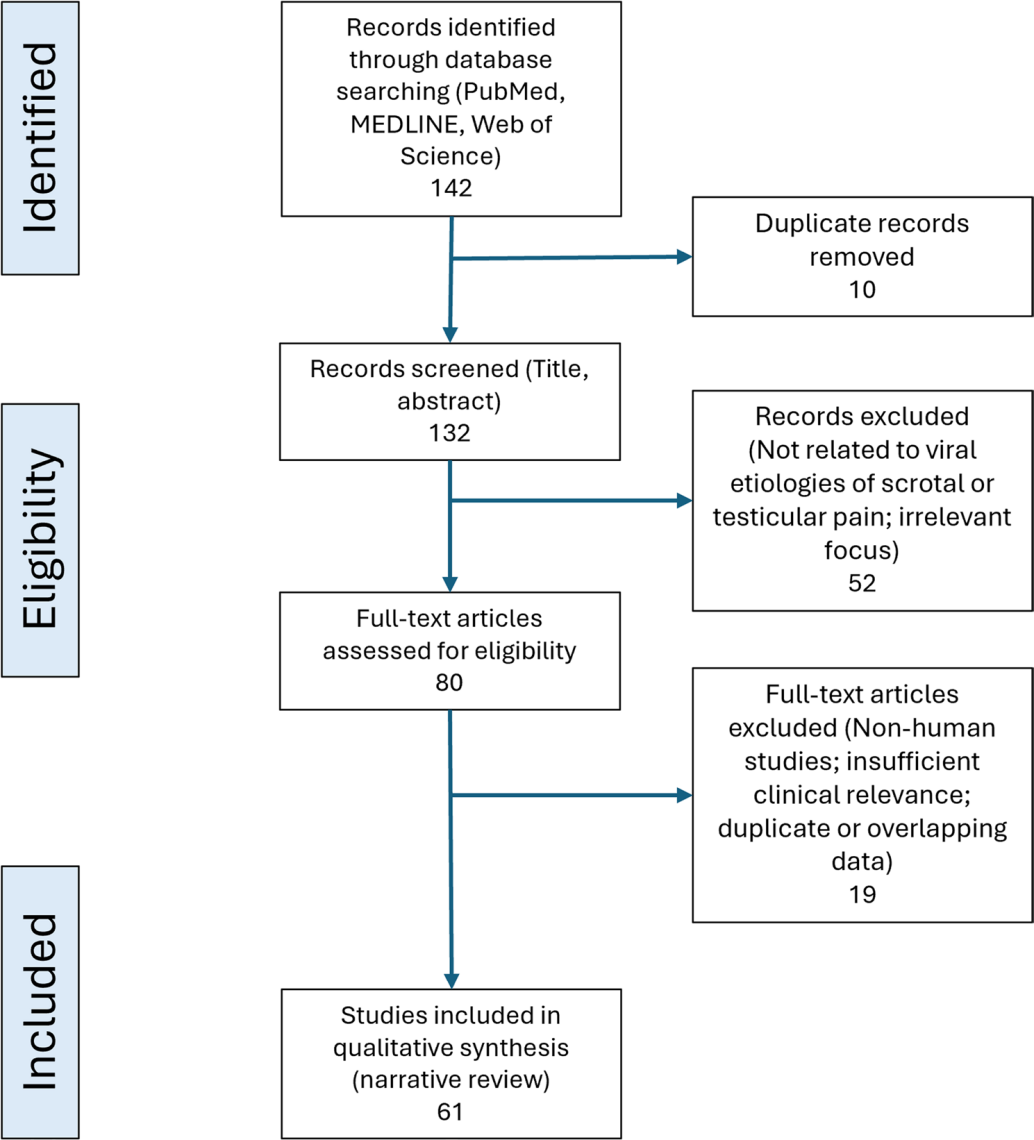
Flow diagram of study identification and selection. *Legend*: The diagram outlines the study identification process: a database search yielded 142 records. After duplicate removal and screening according to predefined criteria, 61 studies were selected for qualitative analysis, illustrating the key filtering steps of the review

We initially identified 142 articles. After screening titles, abstracts, and full texts, 61 articles met our inclusion criteria for qualitative review. A structured overview of the search process, databases, and selection criteria, accompanied by a PRISMA-style flow diagram, provides clarity and transparency (Fig. 1).

## Result & discussion

### Anatomy of the male genital and CSCP pathophysiology

**Fig. 2:**
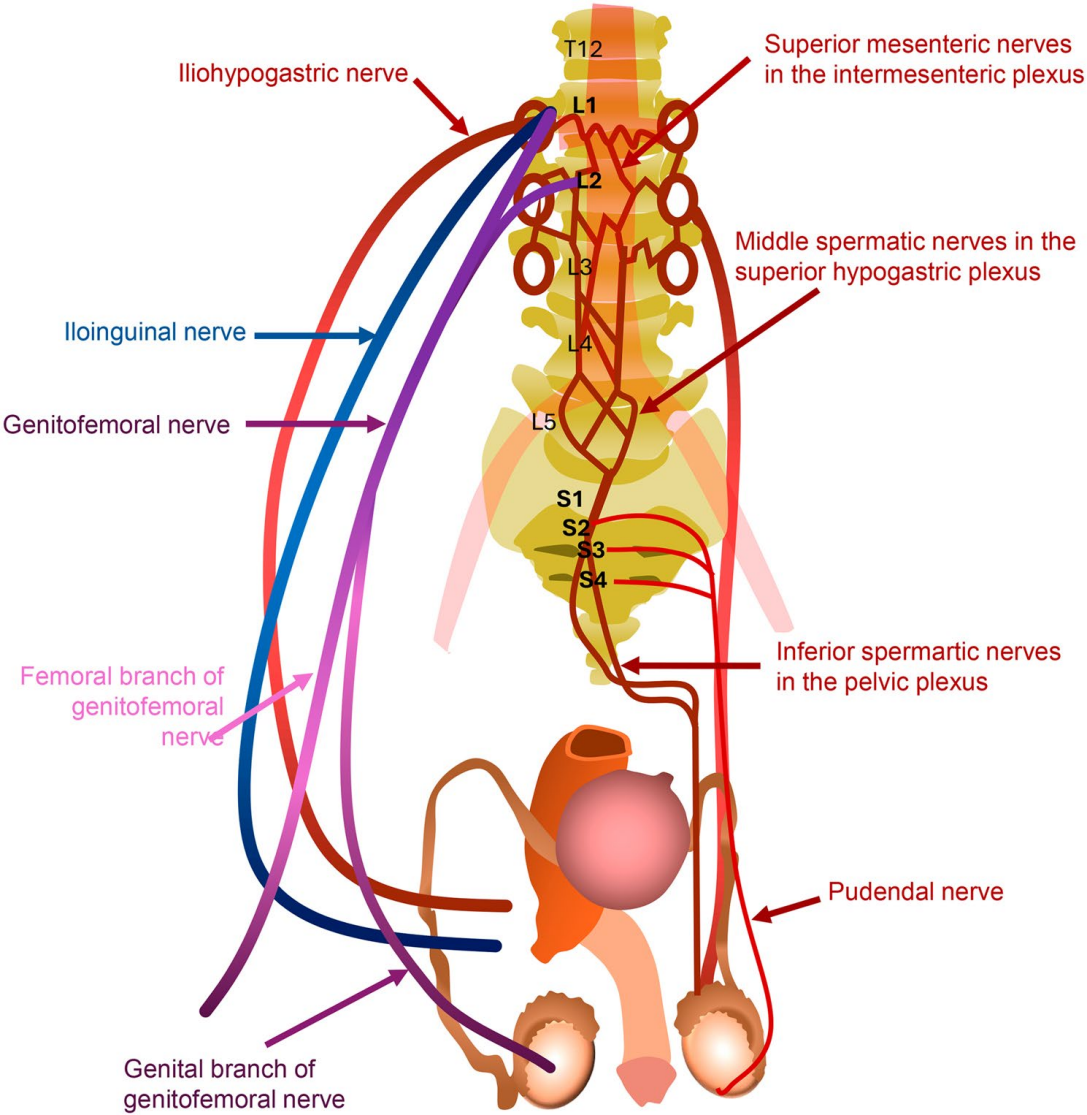
The pathways of somatic and autonomic innervation of the scrotum and testis. *Legend*: On the left side is part of the somatic innervation of the scrotum and testis. The Iliohypogastric nerve (red) originates from L1 and provides sensitivity to the skin over the pubic bone. The Ilioinguinal nerve (blue) originates from L1 and provides sensory innervation to the upper scrotum, penile base, and inner thigh. The genitofemoral nerve (purple) receives its fibers from L1 and L2 nerve roots and is divided into two branches. The femoral branch provides sensitivity to the inner thigh, while the genital branch innervates the cremaster and tunica vaginalis. On the right side, the remainder of the somatic innervation through the pudendal nerve, emerging from S2-S4, is observed, responsible for the innervation of the posterior part of the scrotum. Also on the right side, the superior, middle, and inferior spermatic nerves originated from the renal and intermesenteric plexus, superior hypogastric plexus, and pelvic plexus, respectively. They are responsible for the autonomic innervation of the epididymis and testes [9]

The testes are ellipsoid organs responsible for testosterone synthesis and spermatogenesis. They are housed within the scrotum, a muscular sac that provides protection and regulates temperature to support optimal spermatogenesis [9]. Spermatogenesis occurs in the seminiferous tubules, with spermatozoa subsequently transiting to the epididymis—a highly convoluted duct retroposed to each testis. The epididymis, when uncoiled, measures up to seven meters, and compromises a proximal region formed by the efferent ducts, followed by the head (caput), body (corpus), and tail (cauda). The cauda stores sperm before their passage into the vas deferens and constitutes 5–10% of the ejaculate volume. During ejaculation, sperm mix with secretions from the seminal vesicles and prostate, forming most of the seminal fluid [9–13]. The scrotal contents receive both somatic and autonomic innervation (Fig. 2). Somatic innervation derives from spinal segments L1-L2 and S2-S4 via the iliohypogastric, ilioinguinal, genitofemoral, and pudendal nerves [9]. Sympathetic fibers predominantly arise from T10-L1, while parasympathetic fibers originate from S2-S4. These autonomic fibers course along the testicular vessels and vas deferens, supplying both the epididymis and testes, and are organized as superior, middle, and inferior spermatic nerves [9].

The scrotum and its internal contents share an embryological origin with other organs capable of referred pain, such as the kidneys, ureters, prostate, bladder, and urethra. The afferent fibers from these organs convey signals to the same spinal cord levels (T10-L2), leading to potential overlaps in sensory pathways [4, 8].

### Chronic pain mechanisms in viral infections

Viral infections are implicated in the pathogenesis of chronic neuropathic pain, primarily by inducing an immune response that damages tissues and organs. Cytokine release during this response sensitizes nociceptors and disrupts both the central and peripheral nervous systems. The resulting inflammation may activate aberrant immune mechanisms, leading to chronic pain via pathways such as molecular mimicry, antigen spreading, or bystander activation [14].

Specific viral antigens possess structural similarity to host cell-surface proteins, prompting antibody production by plasma cells that may induce cross-reactivity and subsequent tissue damage [15]. A notable example is chronic peripheral neuropathy associated with immunoglobulin G (IgG) responses following Zika virus or Epstein-Barr virus infection [16]. Additionally, viral infections may elicit autoimmune-mediated chronic pain via antigen dissemination, release of endogenous epitopes secondary to inflammation, or nonspecific activation of autoreactive T and B cells (bystander activation) [17, 18].

Furthermore, viral products can exacerbate chronic pain by facilitating central and peripheral sensitization, a mechanism notably prevalent in the context of retroviruses, such as the human immunodeficiency virus (HIV) and T-cell lymphotropic virus, as well as herpesviruses, including human herpesvirus types 6 and 7, Epstein-Barr virus, and cytomegalovirus [19–21]. Additionally, remnants of viral messenger RNA (mRNA) or proteins may sustain an ongoing immunological response, contributing to the persistence of pain symptoms [22].

### Wallerian degeneration

**Fig. 3:**
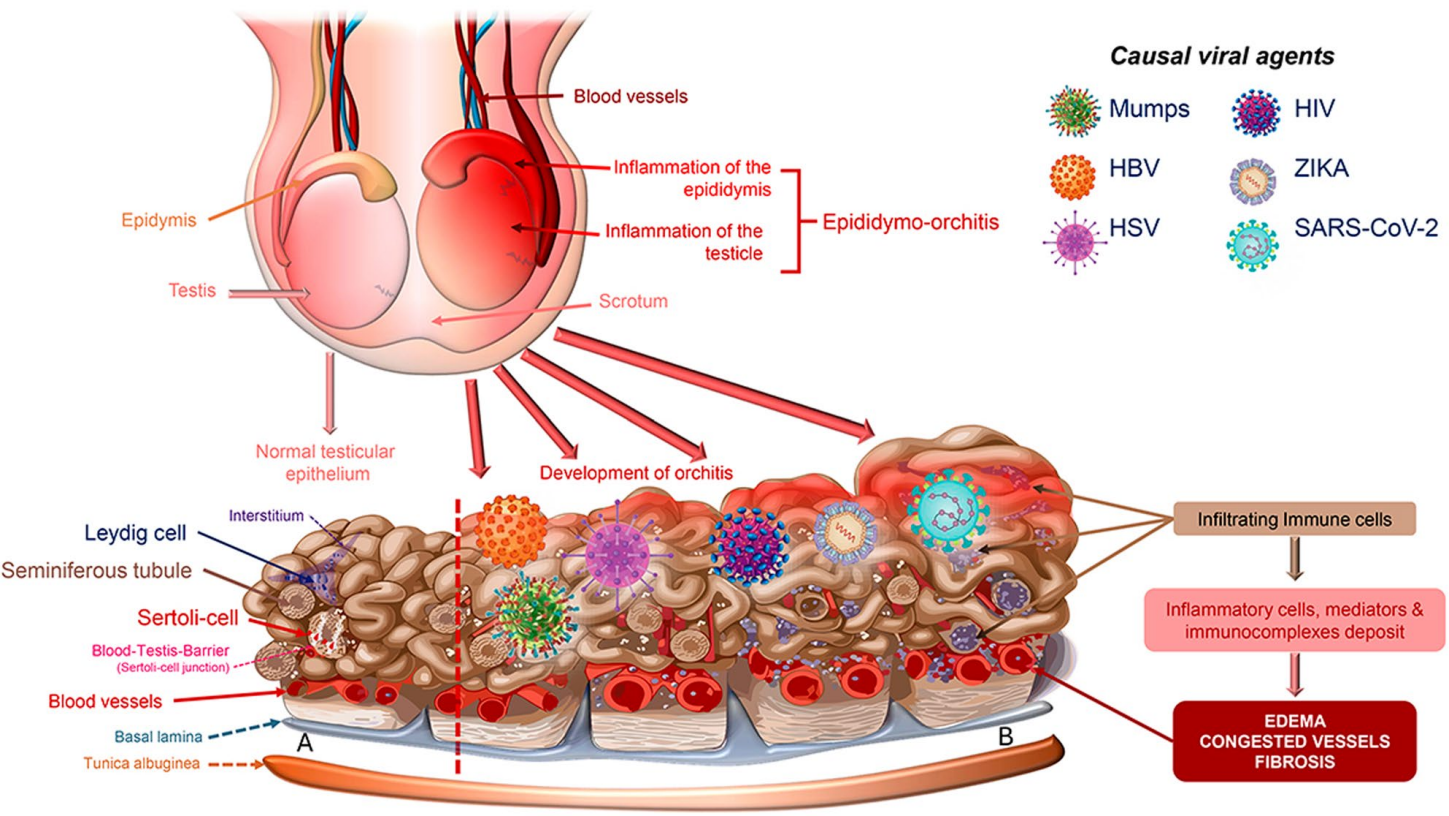
A comprehensive schematic representation of the inflammatory testicular response anticipated during a viral infection. *Legend*: In the healthy testis, the seminiferous tubules are lined by germinal epithelium composed of germ cells at various stages of spermatogenesis and Sertoli cells. Together with intercellular tight junctions, these cells form the hematotesticular barrier, a key physiological barrier providing immune protection to germ cells. The interstitium contains Leydig cells, which produce testosterone, the primary steroid hormone. During viral infections caused by pathogens such as HBV, HSV, HIV, SARS-CoV-2, mumps, and Zika virus, the release of inflammatory mediators is essential for activating the body's defense mechanisms. These mediators induce vasodilation, leading to interstitial edema. Vasodilation also increases vascular endothelial permeability, facilitating the accumulation of a mononuclear inflammatory infiltrate predominantly composed of lymphocytes and macrophages, germ cell degeneration and apoptosis, and structural disorganization of the seminiferous epithelium. This recruitment is crucial for the host's immune response to combat viral pathogens and promote tissue recovery. Additionally, inflammation can compromise the hematotesticular barrier, allowing immune cells to enter the seminiferous tubules and exacerbating testicular damage. In advanced cases, progressive interstitial fibrosis, tubular atrophy, and Leydig cell dysfunction may occur, resulting in primary testicular failure (hypogonadism) and male infertility. *HBV: hepatitis B virus; HSV: herpes simplex virus; HIV: human immunodeficiency virus; SARS-CoV-2: severe acute respiratory syndrome coronavirus 2*

Wallerian degeneration, a physiological mechanism secondary to peripheral nerve injury (Fig. 3), involves establishing an axon-destructive environment to facilitate a debris-free milieu conducive to new axon regrowth [23, 24]. Following nerve injury, calcium influx initiates a process leading to cytoskeletal degradation and axonal degeneration. Immediately after injury, toll-like receptors (TLRs) are activated by binding to ligands released from the injury site. This activation is crucial for signaling through pro-inflammatory cytokines and nearby Schwann cells, increasing vascular permeability and releasing neutrophils, macrophages, and additional pro-inflammatory cytokines to clear the environment and create a supportive milieu for axon regeneration. Within the first 48 h after the injury, Schwann cells play a crucial role in myelin degradation by activating phospholipase A2 (PLA2) and hydrolyzing the myelin phospholipids into phosphatidylcholine. They are later joined mainly by macrophages, which orchestrate the degeneration and debris-clearing process [24]. Although this physiological process is fundamental to axon regeneration, various immune cells, including mast cells, macrophages, neutrophils, and Schwann cells, may contribute to peripheral nerve pain through the numerous pronociceptive mediators released during nerve injury repair [25]. This process is widely accepted as the best plausible theory behind the development and persistence of chronic pain.

## Evidence of viral-induced chronic pain pathways

**Fig. 4:**
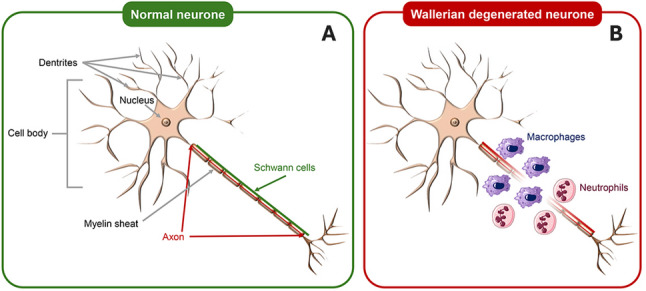
Normal and Wallerian Degenerated Neurons.* Legend*: On the left (**A**) is a typical neuron. Schwann cells surrounding the axons are responsible for axon maintenance and myelin sheath production, which ensures the rapid propagation of electrical impulses. On the right (**B**) is an injured neuron. Toll-like receptors in nearby non-myelinating Schwann cells are activated by binding to ligands at the injury site. These cells participate in myelin degradation. Macrophages and neutrophils subsequently join to complete the clearance process and create an environment suitable for axon regrowth [25]. The timing of regrowth depends on several factors, including the type, intensity, and duration of the insult. Permanent lesions may occur

Studies consistently report that viral infections induce cytokine-mediated sensitization of nociceptors, affecting both peripheral and central pain pathways (Fig. 4). Reports highlight innate immune responses involving interleukin-6 (IL-6), tumor necrosis factor (TNF), interferons, chemokines, and immune cell infiltration [26–30].

Although the direct demonstration of Wallerian degeneration has been well established in bacterial epididymo-orchitis [4, 23], the review found no direct experimental data confirming Wallerian degeneration in chronic scrotal pain following viral orchitis or epididymitis. Surprisingly, the Severe Acute Respiratory Syndrome Coronavirus 2 (SARS-CoV-2) can invade all cells of the reproductive tract. Even spermatozoa generate nuclear DNA-based extracellular traps that rely on cell-free DNA, a phenomenon reminiscent of the extracellular traps (ET) observed in the systemic inflammatory response associated with SARS-CoV-2, which entraps and neutralizes aggressors in a suicidal ETosis-like response [7, 10, 31, 32].

### Viral-specific findings (Fig. 5)

**Fig. 5:**
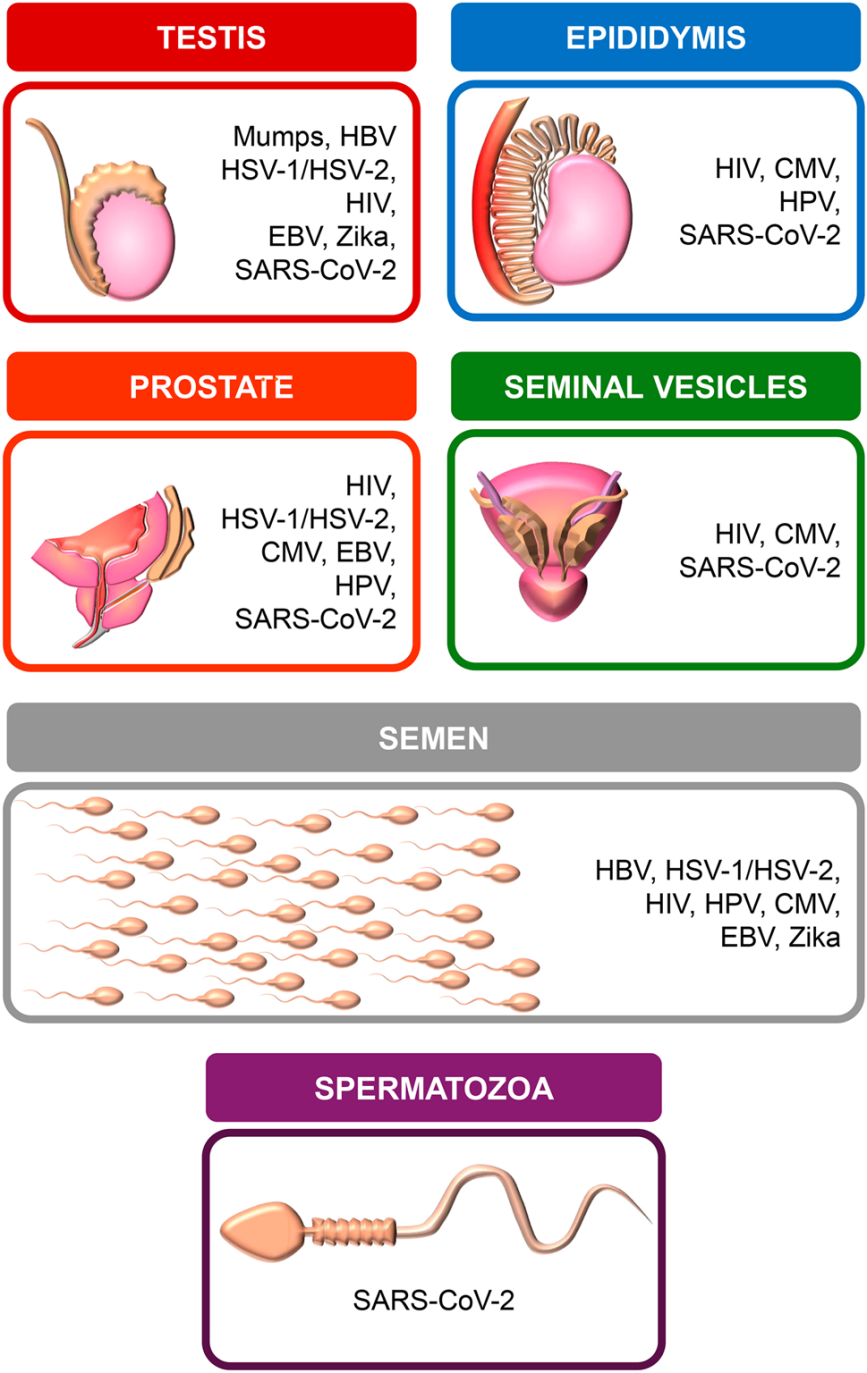
Schematic display of the specific sites and each virus compromising the reproductive system *Legend*: As detailed in Table 1

**Table 1:** Virus characteristics and mechanisms of reproductive tract injury

Virus	Family	Genome	Spread	Reproductive tract injury	References
Mumps virus	*Paramyxoviridae*	*ssRNA (−)*	Saliva or respiratory droplets	Collagen deposit and residual fibrosis (testis)	[27, 33, 34]
HSV-1 and HSV-2	*Herpesviridae*	*dsDNA*	Sexual intercourse	Direct nerve damage, found in prostate and seminal vesicles (cannot infect testis due to BTB)	[10, 35]
HBV	*Hepadnaviridae*	*dsDNA (RT)*	Sexual intercourse	Necrotizing vasculitis due to Polyarteritis nodosa	[10, 11, 36]
HIV	*Retroviridae*	*ssRNA (RT)*	Sexual intercourse	Orchitis, interstitial fibrosis, lymphocyte infiltration	[28, 37, 38]
Zika virus	*Flaviviridae*	*ssRNA (+)*	Infected mosquitoes	Orchitis. Virus RNA found in up to 30 days in Seminal fluid	[11, 39, 40]
SARS-COV2	*Coronaviridae*	*ssRNA (+)*	Inhalation of virus particles	Orchitis fibrin microtrombi, lymphocytic inflammation and seminiferous tubule cellular injury	[11, 32, 41]

Legend - *HSV* Herpes simplex vírus, *HBV* Hepatitis B virus, *HIV* Human immunodeficiency virus, *SARS-CoV-2* Severe acute respiratory syndrome-associated coronavirus type 2. *ssRNA (−)* negative-sense, single-stranded RNA viruses, *ssRNA (+)* positive-sense, single-stranded RNA viruses, *dsDNA* double-stranded DNA viruses, *dsDNA (RT)* double-stranded DNA reverse-transcribing viruses, *ssRNA (RT)* single-stranded RNA reverse-transcribing viruses BTB: blood-testis-barrier

#### Mumps virus

The mumps virus has long been associated with orchitis in post-pubertal humans. In pre-pubertal boys, its clinical impact is often limited to mild parotiditis, with orchitis being the most frequent complication, affecting both testicles in approximately 40% of cases. In 10–30% of cases, it affects both testicles [26]. It is an enveloped RNA virus belonging to the Paramyxoviridae family, with a high affinity for the human testis [42–44]. The main route of mumps virus infection is typically hematogenous spread [43], and the virus has been confirmed in testicular biopsies from infected individuals [45]. The incidence of mumps infection has drastically decreased in the post-vaccine era, with rates varying substantially between countries, from < 0,1/100.000 in the United States and Finland to 10–30/100.000 new cases *per* year in Thailand [46]. A mouse model study was conducted to understand the mechanism of orchitis. The virus induces an innate immune response through TLRs and retinoic acid-inducible gene 1 signaling pathways, leading to the production of pro-inflammatory cytokines, including IL-6, TNF, monocyte chemoattractant protein-1 (MCP-1), C-X-C motif chemokine ligand 10 (CXCL10), and interferons (IFN) such as IFN-α and IFN-β [26, 27].

The diagnosis of mumps orchitis is based on clinical examination. Men usually present with testicular swelling, tenderness, and local redness. Laboratory confirmation is obtained by virus culture, viral RNA detection, or assessment of specific antibody levels [43]. Testicular impacts begin with blood vessel congestion, increased vascular permeability, and lymphocytic invasion, leading to interstitial edema. This typical acute inflammatory state often leads to tissue fibrosis [47] and transiently impairs the testis’ endocrine and reproductive functions. Whether this mumps-induced testicular disease is accompanied by nerve damage due to Wallerian degeneration has not been precisely investigated, as there are no reports in the available literature. However, this is unlikely if we consider that mumps-induced orchitis is a self-limiting condition associated with permanent subfertility/infertility in 30% of patients with orchitis [26].

#### Hepatitis B virus

Hepatitis B is a liver disease caused by the enveloped DNA virus hepatitis B virus (HBV) [36], classified in the Hepadnaviridae family. This virus is transmitted mainly via the sexual route [33] and is notably present in semen. HBV primarily infects hepatocytes in humans, with over 1.5 million new infections each year [10, 11]. HBV is generally transmitted to the reproductive system via the bloodstream. The testis has a notable tropism for endothelial cells and fibroblasts [11, 34, 48]. Polyarteritis nodosa, an uncommon complication of hepatitis B, can affect medium-sized arteries by the deposition of immune complexes with excess antigens, leading to testicular necrotizing vasculitis and chronic testicular pain [10], which could be associated with Wallerian degeneration. Isolation of HBV from the epididymis has not been documented. However, signs of epididymitis have been described during the acute phase of HBV infections [49]. Clinical diagnosis is difficult, as most acute HBV infections are asymptomatic, with epididymitis being a notable exception. The natural course of the infection generally leads to chronic liver infection, eventually progressing to cirrhosis [11]. Laboratory diagnosis of hepatitis B is based on serological tests, principally the detection of hepatitis B surface antigen (HBsAg). Analysis of a combination of antigens enables classification of both acute and chronic stages and determination of vaccination status [36]. Treatment of HBV infection focuses on antiretroviral drugs, notably adenofovir, entecavir, tenofovir, lamivudine, and telbivudine. These drugs are generally prescribed to patients with detectable serum HBV RNA [36].

#### Herpes simplex 1–2 virus

Herpes simplex virus (HSV) is a DNA virus generally transmitted by sexual contact, with two variants. HSV-1 was mainly associated with recurrent ulcers in the perioral area, while HSV-2 was mainly associated with the genital area [11]. Recent studies, however, have demonstrated that both subtypes can cause ulcers in both the oral and genital regions [10, 11, 50]. The herpes virus is transmitted by direct contact with human mucous surfaces. It invades nerve cells and establishes a latent infection that can persist for years, resurfacing on activation [11]. HSV-2 can infect various organs of the genital tract, except for the seminiferous tubules, due to the presence of the blood-testicular barrier [11, 35, 51]. HSV-2 can cause conditions such as urethritis, chronic prostatitis, and epididymitis, which can lead to scrotal discomfort or pain [10, 11, 52, 53]. Diagnosis is usually based on identifying groups of painful vesicles. In the absence of visible lesions, the diagnosis can be confirmed in the laboratory by polymerase chain reaction (PCR) or serological tests to determine past or present viral exposure [11]. Currently, there is no definitive treatment for HSV infections; however, nucleoside analogs such as valaciclovir, acyclovir, or famciclovir are often used to reduce the severity and duration of symptoms [10, 11].

#### Human immunodeficiency virus (HIV)

HIV is a retrovirus that infects human cells, compromising the natural immune system and causing an incurable chronic disease. There are two main viral strains: HIV-1, which is the most widespread and pathogenic, and HIV-2. The virus is transmitted mainly during sexual intercourse, and its initial target is the CD4 T lymphocyte. Testicular pain is a recognized potential symptom of HIV infection, with documented cases of irreversible hypogonadism and severe sperm damage [11, 28, 37, 38]. Although no study has definitively reported that testicular pain presented by HIV patients is systematically associated with the presence of the virus in the testis, the chronic testicular inflammation, mediated by lymphocytic and macrocytic infiltration [28, 29] that accompanies infection, suggests that Wallerian degeneration is probably involved. Laboratory diagnosis is based on antigen-antibody tests. Positive results require confirmation and subtype identification [11].

#### Zika virus

An RNA virus of the Flaviviridae family causes Zika virus infection. Its recent resurgence is mainly due to the proliferation of its vectors in urban areas of deforested regions [11, 39, 40]. Although the mosquito *Aedes aegypti* is the primary vector of the Zika virus, sexual transmission facilitates its spread and is likely responsible for the disease’s severity [11, 39, 40]. To study the mechanisms underlying orchitis observed in many patients, Govero et al. evaluated the testes of mice infected with the Zika virus. They found many CD45 + leukocytes and the absence of ETV5 + cells, indicating loss of the blood-testis barrier, followed by interstitial inflammation and infiltration of F4/80 + macrophages [30].

Zika infection often manifests as a range of nonspecific symptoms, including orchitis, epididymal-orchitis, or prostatitis [39, 40, 54, 55]. These symptoms can eventually become chronic [40], leading to CSCP situations and subfertility mediated by Wallerian degeneration [11]. Due to the predominance of these nonspecific symptoms, diagnosing Zika virus infection can be difficult. When clinical suspicion is high, Zika virus infection can be confirmed by Reverse Transcription Polymerase Chain Reaction (RT-PCR) during the acute phase of the disease [40, 56, 57].

#### SARS-CoV-2 virus

SARS-CoV-2 is a coronavirus, an enveloped RNA virus responsible for the most recent pandemic. Existing plausible evidence points to male sex rather than socioeconomic vulnerabilities as determinants for SARS-CoV-2 infection’s higher death toll and disease severity among males, pointing to a biological factor related to the presence of the testes as a key determinant agent [58, 59]. The virus is transmitted by inhalation and enters the bloodstream *via* the bronchial alveoli by binding to angiotensin-converting enzyme 2 (ACE2) receptors [60, 61]. Because these receptors are abundant in the testis, the testis is susceptible to infection. It is likely to serve as another entry point for the virus in the body, affecting both endocrine and exocrine testicular functions [60]. In 2006, a study conducted in China on six men infected with SARS-CoV, a related coronavirus with a similar mode of infection and strong tropism for ACE2 receptors, reported that the virus was indeed present in the testes and was responsible for an inflammatory response and germ cell destruction [62]. More recently, post-mortem analyses of patients who died of SARS-CoV-2-mediated respiratory distress also revealed the presence of the virus in the testis associated with an inflammatory situation leading to orchitis, with findings of fibrin microthrombi, lymphocytic infiltration, injury to the seminiferous tubule cells, and lower testosterone levels [41, 63–67]. The epididymis also appears to be a potential site of SARS-CoV-2 infection and a potential secondary entry site, with the induction of acute epididymitis in more than 50% of affected individuals, contributing to inflammation [68].

### Proposed hypotheses in viral etiologies of CSCP

The reviewed evidence demonstrates that multiple viral pathogens can infect the male reproductive system and induce varying degrees of inflammation, testicular dysfunction, and scrotal pain. While Wallerian degeneration is well-characterized in bacterial etiologies of CSCP, similar mechanisms have not been conclusively documented in viral etiologies. However, several converging observations support a plausible mechanistic link, such as: (I) viral infections frequently cause significant local inflammation, often breaching protective barriers (e.g., blood-testis barrier in Zika and SARS-CoV-2), (II) cytokine-mediated sensitization of peripheral nerves is a recurrent hallmark of viral disease, (III) chronic inflammatory infiltrates may contribute to long-term nerve irritation or structural injury, and (IV) autoimmune phenomena triggered by molecular mimicry or bystander activation could sustain pain even after viral clearance [14, 30, 32]. Given these overlapping neuropathic and inflammatory pathways, it is reasonable to hypothesize that some cases of persistent scrotal pain following viral infection may involve partial or incomplete Wallerian degeneration or immune-mediated neuropathy, even if direct histological evidence remains lacking. Therefore, this knowledge gap persists as a priority for future research on pain control.

### Potential impact on current clinical practice

Based on these mechanistic insights, several implications for treatment emerge, including: (I) earlier identification of viral etiologies in CSCP, as current diagnostic algorithms prioritize bacterial causes. Incorporating viral screening when patients present with atypical or recurrent scrotal pain may allow earlier targeted management and avoiding unnecessary antibiotic use; (II) anti-inflammatory and neuromodulatory therapies, because viral-induced CSCP may have a neuropathic component [14]; (III) monitoring for hypogonadism, as HIV and SARS-CoV-2 cases show significant associations with reduced testosterone production, supporting routine endocrine evaluation [37, 69]; and (IV) counseling regarding fertility risks, as Zika, mumps, and SARS-CoV-2 infections have all demonstrated impacts on spermatogenesis, so integrating semen analysis in follow-up care may be clinically valuable [11, 70].

Therefore, to operationalize these insights, several actionable steps may be recommended, such as (I) developing standardized diagnostic pathways that incorporate viral testing for patients with acute or chronic pain, especially when bacterial cultures are negative; (II) using multimodal pain management early, emphasizing neuropathic pain protocols when viral etiology is suspected; (III) considering multidisciplinary care, involving andrology, infectious disease and pain specialists for persistent cases; and (IV) promoting vaccination strategies as part of preventive andrological care, given their potential to reduce downstream reproductive sequelae [70, 71].

### Limitations of the study

This narrative review on chronic testicular pain and viral infection is limited primarily by the scarcity of high-quality scientific literature on the topic. Most available studies are small, heterogeneous in design, and often lack standardized diagnostic criteria, which restricts the ability to compare findings across publications. Additionally, the predominance of observational data and case reports weakens causal inference. These gaps highlight the need for well-designed clinical and translational studies to clarify the mechanisms linking viral infection to CSCP and to inform evidence-based management strategies.

## Conclusion

Chronic scrotal content pain is a debilitating condition with many potential causes. While many cases have a clear etiology, a significant proportion remain unexplained, resulting in constant suffering and frustration for the patient. Wallerian degeneration, a process of axonal degeneration triggered by chronic inflammation, has emerged as a possible pathophysiological mechanism underlying CSCP. The clinical consequences of CSCP remain unclear, which explains why so few doctors are investigating this diagnosis and why even fewer publications address it. While bacterial infections have been linked to CSCP, viral infections of the male reproductive system, although often associated with inflammation, have not been extensively studied and are associated with scrotal pain. Examining nerve biopsies from patients with viral infections and correlating them with CSCP could provide valuable insights into the mechanisms underlying these infections and lead to more effective treatments. Understanding the role of viral infections in CSCP is crucial for elucidating its pathogenesis, enhancing diagnostic accuracy, and tailoring treatment strategies to those affected. Further research in this area could reduce the impact of CSCP on the quality of life of these patients worldwide, not only with potential implications in male reproductive health, fertility capacity, both for natural conception and for assisted reproductive techniques success rates, but also for hormonal production by the testis, mitigating the risk factor for the development of hypogonadism later in life.

## Data Availability

No datasets were generated or analysed during the current study.

## Abbreviations

ACE2: angiotensin-converting enzyme 2
CSCP: chronic scrotal content pain
CXCL 10: C-X-C motif chemokine ligand 10
ET: extracellular traps
HBsAg: hepatitis B surface antigen
HBV: hepatitis B virus
HIV: human immunodeficiency virus
HSV: Herpes simplex virus
IFN: interferon
IgG: immunoglobulin G
IL: 6-interleukin-6
MCP: 1-monocyte chemoattractant protein-1
mRNA: messenger RNA
PCR: polymerase chain reaction
PLA2: phospholipase A2
RT-PCR: Reverse Transcription Polymerase Chain Reaction
SARS: CoV-2-Severe acute respiratory syndrome coronavirus 2
TNF: tumor necrosis factor
TLRs: toll-like receptors
