# Correction: Chronic scrotal content pain and viral orchiepididymitis: an overlooked hypothesis linking infection and Wallerian degeneration

**DOI:** 10.1186/s12610-026-00307-3

**Published:** 2026-03-16

**Authors:** Thiago A. Teixeira, Eduardo Zinoni Pato, Maria Christina W. Avellar, Raul Sanchez, Igor V. Coimbra, Joël R. Drevet, Jorge Hallak

**Affiliations:** 1 Androscience, Science and Innovation Center in Andrology, São Paulo, SP Brazil; 2 Division of Urology, Department of Surgery, School of Medicine, University Hospital, Federal University of Amapá, Macapá, Brazil; 3 Division of Urology, Department of Surgery, Hospital das Clinicas - University of São Paulo Medical School, Sao Paulo, Brazil; 4 Department of Pharmacology, Universidade Federal de São Paulo - Escola Paulista de Medicina, São Paulo, Brazil; 5 Faculty of Medicine, Center of Excellence in Translational Medicine–Scientific and Technological Bioresource Nucleus (CEMT–BIOREN), Universidad de La Frontera, Temuco, Chile; 6 Department of Preclinical Sciences, Faculty of Medicine, Universidad de la Frontera, Temuco, Chile; 7 GReD Institute, Faculty of Medicine, EVALSEM, Université Clermont Auvergne, Clermont- Ferrand, France

**Correction: Basic Clin. Androl. 36, 6 (2026)**

**https://doi.org/10.1186/s12610-026-00301-9**

Following publication of the original article [1], the authors identified an error in the legends of Figs. 3 and 4. The incorrect and correct figure legends are given below.

Incorrect Fig. 3 legend:

Normal and Wallerian Degenerated Neurons. *Legend*: On the left is a typical neuron. Schwann cells surrounding the axons are responsible for axon maintenance and myelin sheath production, which ensures the rapid propagation of electrical impulses. On the right is an injured neuron. Toll-like receptors in nearby non-myelinating Schwann cells are activated by binding to ligands at the injury site. These cells participate in myelin degradation. Macrophages and neutrophils subsequently join to complete the clearance process and create an environment suitable for axon regrowth [25]. The timing of regrowth depends on several factors, including the type, intensity, and duration of the insult. Permanent lesions may occur.

Correct Fig. 3 legend:

A comprehensive schematic representation of the inflammatory testicular response anticipated during a viral infection. *Legend*: In the healthy testis, the seminiferous tubules are lined by germinal epithelium composed of germ cells at various stages of spermatogenesis and Sertoli cells. Together with intercellular tight junctions, these cells form the hematotesticular barrier, a key physiological barrier providing immune protection to germ cells. The interstitium contains Leydig cells, which produce testosterone, the primary steroid hormone. During viral infections caused by pathogens such as HBV, HSV, HIV, SARS-CoV-2, mumps, and Zika virus, the release of inflammatory mediators is essential for activating the body’s defense mechanisms. These mediators induce vasodilation, leading to interstitial edema. Vasodilation also increases vascular endothelial permeability, facilitating the accumulation of a mononuclear inflammatory infiltrate predominantly composed of lymphocytes and macrophages, germ cell degeneration and apoptosis, and structural disorganization of the seminiferous epithelium. This recruitment is crucial for the host’s immune response to combat viral pathogens and promote tissue recovery. Additionally, inflammation can compromise the hematotesticular barrier, allowing immune cells to enter the seminiferous tubules and exacerbating testicular damage. In advanced cases, progressive interstitial fibrosis, tubular atrophy, and Leydig cell dysfunction may occur, resulting in primary testicular failure (hypogonadism) and male infertility. *HBV: hepatitis B virus; HSV: herpes simplex virus; HIV: human immunodeficiency virus; SARS-CoV-2: severe acute respiratory syndrome coronavirus 2.*

Incorrect Fig. 4 legend:

A comprehensive schematic representation of the inflammatory testicular response anticipated during a viral infection. *Legend*: **A** In the healthy testis, the seminiferous tubules are lined by germinal epithelium composed of germ cells at various stages of spermatogenesis and Sertoli cells. Together with intercellular tight junctions, these cells form the hematotesticular barrier, a key physiological barrier providing immune protection to germ cells. The interstitium contains Leydig cells, which produce testosterone, the primary steroid hormone. **B** During viral infections caused by pathogens such as HBV, HSV, HIV, SARS-CoV-2, mumps, and Zika virus, the release of inflammatory mediators is essential for activating the body’s defense mechanisms. These mediators induce vasodilation, leading to interstitial edema. Vasodilation also increases vascular endothelial permeability, facilitating the accumulation of a mononuclear inflammatory infiltrate predominantly composed of lymphocytes and macrophages, germ cell degeneration and apoptosis, and structural disorganization of the seminiferous epithelium. This recruitment is crucial for the host’s immune response to combat viral pathogens and promote tissue recovery. Additionally, inflammation can compromise the hematotesticular barrier, allowing immune cells to enter the seminiferous tubules and exacerbating testicular damage. In advanced cases, progressive interstitial fibrosis, tubular atrophy, and Leydig cell dysfunction may occur, resulting in primary testicular failure (hypogonadism) and male infertility. *HBV: hepatitis B virus; HSV: herpes simplex virus; HIV: human immunodeficiency virus; SARS-CoV-2: severe acute respiratory syndrome coronavirus 2*.

Correct Fig. 4 legend:

Normal and Wallerian Degenerated Neurons. *Legend*: On the left (A) is a typical neuron. Schwann cells surrounding the axons are responsible for axon maintenance and myelin sheath production, which ensures the rapid propagation of electrical impulses. On the right (B) is an injured neuron. Toll-like receptors in nearby non-myelinating Schwann cells are activated by binding to ligands at the injury site. These cells participate in myelin degradation. Macrophages and neutrophils subsequently join to complete the clearance process and create an environment suitable for axon regrowth [25]. The timing of regrowth depends on several factors, including the type, intensity, and duration of the insult. Permanent lesions may occur.

The original article [1] has been corrected.
